# Detection of PrP^BSE^ and prion infectivity in the ileal Peyer’s patch of young calves as early as 2 months after oral challenge with classical bovine spongiform encephalopathy

**DOI:** 10.1186/s13567-017-0495-5

**Published:** 2017-12-19

**Authors:** Ivett Ackermann, Anne Balkema-Buschmann, Reiner Ulrich, Kerstin Tauscher, James C. Shawulu, Markus Keller, Olanrewaju I. Fatola, Paul Brown, Martin H. Groschup

**Affiliations:** 1 Institute of Novel and Emerging Infectious Diseases, Friedrich-Loeffler-Institut, Greifswald-Insel Riems, Germany; 2 Department of Experimental Animal Facilities and Biorisk Management, Friedrich-Loeffler-Institut, Greifswald-Insel Riems, Germany; 3 National Institute of Neurological Diseases and Stroke, National Institutes of Health, Bethesda, MD USA

## Abstract

In classical bovine spongiform encephalopathy (C-BSE), an orally acquired prion disease of cattle, the ileal Peyer’s patch (IPP) represents the main entry port for the BSE agent. In earlier C-BSE pathogenesis studies, cattle at 4–6 months of age were orally challenged, while there are strong indications that the risk of infection is highest in young animals. In the present study, unweaned calves aged 4–6 weeks were orally challenged to determine the earliest time point at which newly formed PrP^BSE^ and BSE infectivity are detectable in the IPP. For this purpose, calves were culled 1 week as well as 2, 4, 6 and 8 months post-infection (mpi) and IPPs were examined for BSE infectivity using a bovine PrP transgenic mouse bioassay, and for PrP^BSE^ by immunohistochemistry (IHC) and protein misfolding cyclic amplification (PMCA) assays. For the first time, BSE prions were detected in the IPP as early as 2 mpi by transgenic mouse bioassay and PMCA and 4 mpi by IHC in the follicular dendritic cells (FDCs) of the IPP follicles. These data indicate that BSE prions propagate in the IPP of unweaned calves within 2 months of oral uptake of the agent.

**Electronic supplementary material:**

The online version of this article (10.1186/s13567-017-0495-5) contains supplementary material, which is available to authorized users.

## Introduction

Transmissible spongiform encephalopathies (TSEs), or prion diseases, are fatal neurodegenerative disorders including scrapie, bovine spongiform encephalopathy (BSE) and chronic wasting disease (CWD) in animals and Creutzfeldt-Jakob disease (CJD) in humans. TSEs are characterised by the accumulation of an abnormal isoform of the prion protein (PrP^TSE^), resulting from conversion of a host-encoded cellular membrane-bound glycoprotein (PrP^C^) [1]. In classical bovine spongiform encephalopathy (C-BSE) of cattle, prions spread centripetally mainly via the autonomic nervous system from the gut to the central nervous system, primarily utilizing sympathetic and to a lesser extent parasympathetic structures, while the spinal cord constitutes an additional route [2]. Specified risk materials (SRM), which possibly contain BSE infectivity in incubating cattle, have been banned from manufacturing of food, health products and animal feedstuff, in order to minimize the BSE exposure risk for humans and cattle. Currently the list of SRM from bovines of all ages, as defined in the Regulation (EC) No 999/2001 (consolidated version as of 12.2016) includes the last four metres of the small intestine (distal ileum), the caecum, the mesentery and the tonsils in countries having a controlled BSE risk status, while no restrictions regarding the intestine apply for negligible risk countries.

The ileal Peyer’s patch (IPP), representing the gut-associated lymphoid tissue (GALT) of the ileum, has been postulated to function as the primary site of entry for the BSE agent in cattle, as PrP^BSE^ and BSE infectivity have been detected from 4 mpi in this location [3–6]. The IPP is a packed lymphatic structure in young cattle with a maximal extension of about 4 m length at 12–18 months of age, which undergoes an involution that is completed at approximately 2 years of age (with a IPP length of about 0.3 m) [7]. In previous studies PrP^BSE^ has been detected in the IPP follicles from 4 mpi and in the enteric nervous system (ENS) of the distal ileum as early as 16 mpi [3, 4]. In those earlier reports, neither infectivity nor PrP^BSE^ was detectable prior to 4 mpi, and the earliest sampled time point 1 mpi was tested negative [3, 4]. However, the time point of initial accumulation of PrP^BSE^ and BSE infectivity in the IPP was not yet determined.

In C-BSE pathogenesis studies performed thus far, cattle at 4–6 months of age were challenged with the BSE agent [8, 9]. However, there are strong indications that age has an impact on susceptibility to prion infections in sheep and rodents [10–12], so that an age-related evaluation appears to be crucial. Susceptibility was decreased in weaned sheep (3 and 6 months old and adult) orally challenged with BSE, as compared to 2–3 week-old lambs [10]. Epidemiological data indicate that calves up to 6 months of age had a higher infection risk during the recent BSE epidemic [13], but it was not determined whether this was due to a higher susceptibility or to a different feeding management in this young age group. An experimental BSE infection of young unweaned calves has never been performed before.

Here, we present results of an early C-BSE pathogenesis study with an emphasis on the entry port for BSE prions, the IPP, of young unweaned calves aged 4–6 weeks at the time of oral challenge. To determine the earliest time point of PrP^BSE^ and infectivity accumulation in the IPP after oral infection, we examined samples collected at incubation periods as early as 1 week as well as 2, 4, 6 and 8 months post-infection. This choice of animals, together with the application of ultra-sensitive methods for the detection of bovine PrP^BSE^/prion infectivity, add new insights to the early BSE pathogenesis in the intestine by showing BSE infectivity in the ileum of cattle earlier than heretofore reported.

## Materials and methods

### Animals

To warrant the worst case scenario of the supposedly highest prion susceptibility, unweaned calves aged 4–6 weeks were chosen for oral BSE challenge. 20 unweaned Simmental mixed male and female calves were orally challenged with classical BSE using a 100 g dose of a brainstem homogenate pool of >50 clinically diseased cattle. Using a syringe with a metal applicator, 200 mL of a 50% brainstem in a 5% sucrose solution were administered on the base of the tongue. In addition, two control calves were orally inoculated with a BSE-negative brainstem homogenate pool derived from healthy slaughtered cattle.

18 of the 20 challenged calves were killed and necropsied at scheduled time points of 1 week as well as 2, 4, 6 and 8 months post inoculation. During necropsy, a wide range of tissues from the central and peripheral nervous system, the lymphoreticular system and the digestive tract were sampled under TSE-sterile conditions.

As positive controls, two of the challenged calves were kept to monitor the development of clinical BSE symptoms. From 24 months post infection, a standardised neurological examination was performed every 4 weeks as described elsewhere [14]. This starting point was chosen since 24 months post infection was the earliest time point when PrP^BSE^ was found in the brainstem of cattle before [2, 8].

The experimental animals were sourced from three closed herds (managed in accordance to the Scientific Opinion “BSE Negligible risk (closed) bovine herds” adopted by the Scientific Steering Committee at its meeting of 22–23 July 1999) with no history of BSE. These closed herds were established in 1993, 2004 and 2007. All infected cattle were housed in a free-ranging group. The animals were fed twice a day, as determined by conditions in the BSL-3** facility, and were nourished with hay, hay cobs, crimped oats and concentrates at the appropriate rates. Negative control cattle were housed separately in another stable under comparable conditions.

#### Ethical statement

The challenge experiments in cattle and mice described in this manuscript were approved by the competent authority of the Federal State of Mecklenburg-Western Pomerania, Germany on the basis of national and European legislation, namely the EU council directive 2010/63/EU for the protection of animals used for experiments (File Number: 7221.3-1.1-037/13).

### Tissue samples

The ileal Peyer’s patches (IPP) were examined from 18 challenged calves that were sacrificed between 1 week and 8 months post challenge as well as from the two negative controls (*n* = 20). All samples were analysed by immunohistochemistry (IHC) and protein misfolding cyclic amplification (PMCA) for PrP^BSE^ depositions and by transgenic Tgbov XV mouse bioassay for the detection of BSE infectivity. During necropsy the IPP was sampled as described before [3]. IPP samples were divided into two halves, while one half was fixed in formalin to perform the IHC analyses and the other half was stored frozen for the bioassay and PMCA studies. Nearly all ileal tissue samples (*n* = 19) contained Peyer’s patches (PP), while the tissue sample taken from animal WAIT 16 (2 mpi) (*n* = 1) surprisingly did not contain any PP, therefore the ileocaecal junction of this animal was additionally examined by IHC.

However, some IPP samples only contained low numbers of IPP follicles. To reach a statistically calculated minimal number of follicles to be examined by IHC per animal, initially frozen tissue samples from two animals, WAIT 15 and WAIT 16 (2 mpi, *n* = 2) were fixed in formalin and examined. In the case of WAIT 16, we prepared the frozen IPP sample for IHC that was directly adjacent to the tissue used to prepare the bioassay inoculum, in order to evaluate if the IPP follicles had been included in the inoculum. By using this approach, the histological examination of the frozen IPP sample of WAIT 16 proved the presence of IPP follicles in the bioassay inoculum.

### Immunohistochemistry (IHC) and histopathological examination

With some modifications, tissue samples were processed as described earlier [2, 15]. The IPP samples were fixed in 4% neutral buffered formaldehyde for at least 2 weeks. The fixed tissues were cut into blocks, dehydrated and embedded in paraffin wax according to standard histopathological methods.

Sections (3 µm) were prepared and mounted on SuperFrost Plus slides (Thermo Fisher Scientific Gerhard Menzel, Braunschweig, Germany). A serial section procedure [3] was used to examine five different levels per block with a plane distance of about 30 µm. Thus, a depth of approximately 195 µm per block was attained. For IPP samples from positive control animals (WAIT 01 and 04, *n* = 2) one level per block was examined, as this was sufficient to analyse the aimed 99 follicles.

For the histopathological examination, a hematoxylin–eosin staining was performed according to standard histological methods. A Periodic Acid Schiff (PAS) reaction was accomplished according to standard methods, by using periodic acid and Schiff reagent from a Periodic Acid Schiff (PAS) Hotchkiss-McManus staining kit (DDKItalia, Vigevano, Italy).

For the IHC staining, two PrP-specific monoclonal antibodies (mAbs) were used, which were mAb 6C2 (recognizes epitope HVAGAAAA—residues 122–129 of the bovine PrP; Wageningen Bioveterinary Research, Lelystad, Netherlands) and mAb F99 (F99/97.6.1, recognizes epitope QYQRES—residues 217–231 of the ovine PrP [16, 17]; VMRD, Pullman, USA). After establishing the optimal dilution for these two mAbs as well as for mAb 12F10 [18], we directly compared the diagnostic sensitivities of these three antibodies, which revealed similar sensitivities of mAb 6C2 and mAb F99 (while mAb 12F10 gave a slightly weaker signal). Hence, IHC staining was routinely performed using mAb 6C2. In some instances, additional sections were stained with mAb F99 for verification of positive ENS staining or inconclusive results.

After rehydration, sections were pretreated with 98% formic acid for 15 min and rinsed in tap water for 5 min. Endogenous peroxidase was blocked with 3% H_2_O_2_ in distilled water for 30 min. Before the PrP-specific primary antibodies mAb 6C2 or mAb F99 were applied, sections underwent hydrated autoclaving in citrate buffer (pH 6.0) at 121 °C for 20 min. Inhibition of endogenous biotin was accomplished using an avidin/biotin blocking kit (Vector Laboratories, Burlingame, USA) by incubating the sections with an Avidin D solution and a biotin solution for 15 min each, with a short rinse in TBS between these steps. The monoclonal antibodies were diluted 1:1400 (6C2, stock solution 1 mg/mL) and 1:1400 (F99, stock solution 1 mg/mL) in goat serum and incubated for 30 min at room temperature. On negative control sections, the anti-PrP mAb 3F4 (dilution 1:800, stock solution 1 mg/mL, CHEMICON International, Temecula, USA) was applied, which does not bind to bovine PrP [15] but efficiently detects human, feline and hamster PrP [19]. Visualisation was achieved using the Vectastain^®^ Elite ABC Kit (Vector Laboratories, Burlingame, USA) and diaminobenzidine (DAB substrate kit, Vector Laboratories) [15]. Finally, sections were counterstained with Mayer’s hematoxylin, dehydrated and mounted with Entellan (Merck, Darmstadt, Germany).

All slides were analysed by light microscopy and Peyer’s patch follicles were counted to determine the percentage of positive IPP follicles.

#### Determination of the sampling size of IPP follicles

In immunohistochemically stained sections, all follicles of the ileal Peyer’s patch (IPP) were counted to determine the percentage of positive IPP follicles for each sample. The number of follicles to be analysed was calculated using a unilateral 95% confidence interval. For IPPs of calves that were assumed to be negative by IHC (0 mpi, 2 mpi and negative controls, *n* = 6, *P* < 0.001) the aim was to analyse a total number of 2995 follicles. For animals for which positive results were to be expected as deduced from earlier comparable experiments [3, 5, 6] (4, 6 and 8 mpi, *n* = 14, *P* < 0.002), a total number of 1497 or more follicles was aimed at. As shown by an earlier study, the proportion of positive follicles is increased with age [5] and for animals at 30 mpi P < 0.03 can be presumed. Therefore for positive control animals (WAIT 01, 36 mpi and 04, 35 mpi; *n* = 2, *P* < 0.03) a number of 99 follicles was aimed to be analysed.

### Tgbov XV mouse bioassay

Tissue sample which clearly contained IPP of 18 infected and two negative control calves were examined by mouse bioassay, using transgenic mice over-expressing bovine PrP [20]. 20 Tgbov XV mice per group were intracerebrally inoculated using 30 µL of a 10% (w/v) tissue homogenate diluted in sterile 0.9% saline solution. Tissue homogenates were tested on blood agar plates and initially only five mice of each group were inoculated to confirm the absence of bacterial or toxic components in the inoculum. 15 more mice were inoculated if bacterial cultivation did not reveal any colony-forming units (cfu), and if the initially inoculated mice survived for at least 1 week. Inocula were heat-treated for 10 min at 70 °C, in case of colony-forming units on blood agar after at least 72 h incubation at 37 °C, or death of animals among the initially challenged five mice. All mice were monitored for the onset of clinical signs at least twice per week. Animals showing at least two clinical symptoms indicative of a BSE infection, such as hind limb paresis, abnormal tail tonus, behavioural changes and weight loss over several consecutive days [20] were sacrificed and brain samples were taken for diagnostic evaluation. The brains were analysed for the presence of PrP^BSE^ by digestion with 50 µg/mL Proteinase K at 55 °C for 1 h followed by Western blot using mab L42 at a concentration of 0.4 μg/mL (r-biopharm, Darmstadt, Germany) as detection antibody. Any inconclusive results were verified by PTA immunoblotting [21]. Results of mice incubating at least 100 dpi were included in the interpretation.

#### End-point titration of brainstem pool

To determine the infectivity load (LD_50_) of the brainstem pool used as the inoculum for the calves, an end-point titration experiment in Tgbov XV mice was performed as described before [20]. Groups of eight mice each were inoculated with different dilutions of the brainstem pool: 10^−3.4^, 10^−4.1^, 10^−4.8^, 10^−5.5^, 10^−6.2^, 10^−6.9^, 10^−7.6^ and 10^−8.3^. Mice were monitored and analysed as described above.

The LD_50_-titer with a 95% confidence interval was estimated employing logistic regression using R software, Version 2.15.2, with doBy, Version 4.5-5 [22, 23].

### Protein misfolding cyclic amplification (PMCA)

The PMCA protocol was applied as described earlier [24, 25] with some modifications. Briefly, brain tissue from Tgbov XV transgenic mice [26] was used as the PrP^C^ source for the PMCA reaction. Brain samples were homogenized to a concentration of 10% (w/v) in PMCA conversion buffer [PBS containing 150 mM NaCl, 1.0% Triton X-100, 5 mM EDTA and Complete Protease Inhibitor Cocktail (Roche, Mannheim, Germany)] to prepare the substrate solution. The template for the positive control PMCA reaction was a 10% (w/v) homogenate of bovine brain tissue in PBS. Serial dilutions were prepared in substrate solution. Analyte tissue samples were homogenized at 10% (w/v) in 0.9% saline solution. A 10 µL aliquot of the analyte homogenate was suspended in 90 µL Tgbov XV brain substrate and transferred into 0.5 mL reaction tubes. A brain sample of a confirmed BSE negative cattle served as a negative control, while dilutions of a confirmed BSE positive brain (10^−3^, 10^−6^ and 10^−9^) were used as positive controls in each experiment. All controls and all samples were analysed in duplicate.

The tubes were placed into a thin-walled adaptor and put on a microplate module of a sonicator (Sonicator 3000, Misonix, Farmingdale, USA). Samples were exposed to 48 cycles of sonication for 20 s each at a potency of 210–250 W (level 8), followed by a 30 min incubation. The reaction was carried out in a 35 °C incubator. After the first round of 48 cycles, samples were diluted 1:10 in fresh substrate solution and subjected to a second round of 48 cycles, which was again followed by a 1:10 dilution and a third PMCA round.

1% Sarkosyl and 0.06% SDS final concentrations were added to the PMCA products prior to incubation with Proteinase K (150 µg/mL), for 60 min at 55 °C. Finally, the samples were analysed by Western blotting as described above. The experiment was considered valid if at least the 10^−3^ and 10^−6^ dilutions were clearly identified as positive, and the negative control gave a negative result. The obtained results for the IPP samples were interpreted as follows: a clear PrP^BSE^ signal in all three PMCA rounds was interpreted as +++ positive, a signal in the second and third PMCA round was interpreted as ++ positive, and a signal only in the third PMCA round was interpreted as + positive.

## Results

### Clinical symptoms of positive control cattle

The 18 challenged calves and two negative controls did not develop any clinical signs of BSE during the duration of the predetermined incubation periods between 1 week and 8 months post infection (Table 1).

**Table 1:** Results of ileal Peyer’s patch samples with reference to BSE status and Obex IHC results

Months pi	Animal ID	Status	Obex (IHC)	Ileal Peyer’s patch		
				IHC	PMCA	Tgbov XV mouse bioassay#
0 (*n* = 2) (= 1 week pi)	WAIT 17	Preclinical	neg.	neg.	−	7/19, 567 ± 43
	WAIT 18	Preclinical	neg.	neg.	−	2/21, 424 ± 76
2 (*n* = 2)	WAIT 15	Preclinical	neg.	neg.	+	12/20, 537 ± 42
	WAIT 16	Preclinical	neg.	neg.	−	3/21, 525 ± 47
				ICJ: +s.pl.*	ICJ: −	
4 (*n* = 6)	WAIT 11	Preclinical	neg.	pos.	–	3/20, 567 ± 58
	WAIT 12	Preclinical	neg.	pos.	+ (+)	17/20, 396 ± 28 (~)
	WAIT 13	Preclinical	neg.	pos.	+	14/20, 449 ± 19 (~)
	WAIT 14	Preclinical	neg.	pos.	++	20/20, 362 ± 12
	WAIT 19	Preclinical	neg.	inconcl.	+	10/20, 473 ± 17 (~)
	WAIT 20	Preclinical	neg.	pos.	++ (+)	20/20, 315 ± 11
6 (*n* = 6)	WAIT 05	Preclinical	neg.	pos.	++	19/20, 307 ± 15
	WAIT 06	Preclinical	neg.	pos.	++	19/20, 368 ± 16
	WAIT 07	Preclinical	neg.	pos.	+ (+)	19/20, 383 ± 15 (~)
	WAIT 08	Preclinical	neg.	pos.	++ (+)	20/20, 319 ± 8
	WAIT 09	Preclinical	neg.	pos.	++	20/20, 366 ± 14
	WAIT 10	Preclinical	neg.	pos.	+	19/20, 383 ± 16
8 (*n* = 2)	WAIT 02	Preclinical	neg.	neg.	++ (+)	18/20, 401 ± 21
	WAIT 03	Preclinical	neg.	pos.	+++	18/20, 396 ± 12
8 (*n* = 2)	WAKT 01	Negative control	neg.	neg.	−	0/20, > 730
	WAKT 02	Negative control	neg.	neg.	−	0/20, > 730
35 (*n* = 1)	WAIT 04	3 (Positive control)	pos.	pos.	+++	Not done
36 (*n* = 1)	WAIT 01	2 (Positive control)	pos.	pos.	++ (+)	Not done

pi: post infection; ICJ: ileocaecal junction; IHC: immunohistochemistry; neg.: negative; pos.: positive; inconcl.: inconclusive; +s.pl.*: positive submucosal plexus (only seen with mAb 6C2); PMCA: protein misfolding cyclic amplification; +: positive in the third round; ++: positive in the second and third round; +++: positive from the first round; (+): weak positive; −: negative; # positive/inoculated mice and mean incubation time in days ± standard error of the mean (SEM); (~) bioassay is ongoing: so far positive/inoculated mice and their mean incubation time in days ± standard error of the mean (SEM).

We monitored the two positive control cattle (WAIT 01 and 04) monthly for the development of clinical symptoms of a BSE infection by performing a neurological examination, starting at 24 mpi. From 30 mpi, both animals’ body condition score decreased continuously and mild emaciation was observed at 34 mpi. At 32 mpi one positive control animal (WAIT 04) showed mild symptoms suspicious for the early stage of the disease, such as a startled over-reaction to acoustic stimuli, as well as fear from stepping over obstacles on the ground such as a bar and was therefore judged as probable BSE. At 34 mpi (status 3, definitive BSE), this animal seemed nervous during the entire time of the examination, as shown by teeth grinding, frequent licking of the muzzle and nervous ear twitching. In addition to the symptoms described above, the animal displayed hypersensitivity to optic and tactile stimuli, primarily when touching the head. We sacrificed the animal at 35 mpi. From 36 mpi the second positive control animal (WAIT 01) showed BSE-related clinical symptoms characterised by hypersensitivity to tactile stimuli particularly at the hind limbs, pelvic region and back as well as a mild over-crossing of the hind limbs in motion (status 2, probable BSE). Shortly after the onset of illness, the animal was killed and necropsied for animal welfare reasons. For both animals we confirmed a BSE infection by immunohistochemical analysis of the obex and found their IPPs to be positive by IHC and PMCA (Tables 1 and 2, Additional file 1).

**Table 2:** Detailed results of immunohistochemical analyses for ileal Peyer’s patch samples

Months pi	Animal ID	Immunohistochemistry (IHC) of ileal Peyer’s patch				
		No. pos. foll./ Total no. foll.	No. pos. foll. In %	TBM	FDC	ENS
0 (*n* = 2) (1 week pi)	WAIT 17	0/3288	0	−	−	−
	WAIT 18	0/3636	0	−	−	−
2 (*n* = 2)	WAIT 15	0/3837	0	−	−	−
	WAIT 16	0/5136	0	−	−	−
		Ileocaecal junction (ICJ):		ICJ: −	ICJ: −	ICJ: + s.pl.*
		0/887	0			
4 (*n* = 6)	WAIT 11	2/1976	0.10	Fine granular	(+)	−
	WAIT 12	3/1820	0.16	Punctate/fine granular	−	−
	WAIT 13	24/2873	0.84	Fine/coarse granular	(+)	−
	WAIT 14	19/1993	0.95	Fine/coarse granular	(+)	−
	WAIT 19	1*/3986	inconcl.	Fine granular	−	−
	WAIT 20	89/2088	4.26	Fine/coarse granular	+	−
6 (*n* = 6)	WAIT 05	25/2283	1.10	Fine/coarse granular	+	−
	WAIT 06	43/1997	2.15	Fine/coarse granular	−	−
	WAIT 07	7/1783	0.39	Fine granular	−	−
	WAIT 08	85/1622	5.24	Fine/coarse granular	+	−
	WAIT 09	6/1473	0.41	Punctate/fine granular	−	−
	WAIT 10	1/2207	0.05	Punctate/fine granular	−	−
8 (*n* = 2)	WAIT 02	0/2203	0	–	−	−
	WAIT 03	17/1446	1.18	Fine/coarse granular	−	−
8 (*n* = 2)	WAKT 01	0/3925	0	–	−	−
	WAKT 02	0/2911	0	–	−	−
35 (*n* = 1)	WAIT 04	9/184†	4.89	Fine/coarse granular	−	−
36 (*n* = 1)	WAIT 01	8/131†	6.11	Coarse granular	+	++ m.pl.

pi: post-infection; No.: number; foll.: follicles; inconcl.: inconclusive; *: only seen with mAb 6C2; †: in one level of the block; TBM: tingible body macrophages; −: negative; FDC: follicular dendritic cells; (+): weak net-like staining pattern; +: clear dendritic network; −: negative; ENS: enteric nervous system, positive labelling in submucosal (s.pl.) or myenteric (m.pl.) plexus; +: 0–4 positive foci, ++: 5–10 positive foci; −: negative.

### Histopathology

Animals sacrificed at the predetermined time points showed neither BSE-related clinical signs nor histopathological changes in the obex region. Systematic histopathological examination revealed only a mild lymphohistiocytic and eosinophilic infiltration in the lamina propria mucosae in almost all calves, including the negative controls, possibly due to a subclinical infection with *Eimeria* spp., as indicated by scattered schizonts, gamonts and oocysts in the lamina propria mucosae of some animals.

### Immunohistochemical detection of PrP^BSE^ depositions

We detected PrP^BSE^ in the ileal Peyer’s patches (IPP) of 12 out of the 18 calves between 1 week and 8 months post challenge (Table 2).

The earliest PrP^BSE^ deposition was observed in IPP follicles of animals sacrificed 4 mpi. Interestingly, the quantity of PrP^BSE^ positive follicles was independent of the age of the animal or the time point after infection. In 5 of the 12 positive calves, we saw depositions only in individual or few follicles, i.e. in less than 0.5% of the examined follicles (WAIT 11, 12; 4 mpi and WAIT 07, 09, 10; 6 mpi). In contrast, other animals had accumulated PrP^BSE^ in between 0.84% and 1.18% of the follicles (WAIT 13, 14; 4 mpi, WAIT 05; 6 mpi and WAIT 03; 8 mpi). For individual calves, we determined higher positive follicle numbers, with 2.15% (WAIT 06; 6 mpi), 4.26% (WAIT 20; 4 mpi) and 5.24% (WAIT 08; 6 mpi). Surprisingly, the IPP of one calf (WAIT 02; 8 mpi) was negative in IHC and for another animal (WAIT 19; 4 mpi) the result remained inconclusive as a single follicle was detected positive with mAb 6C2 and negative with mAb F99.

**Figure 1:**
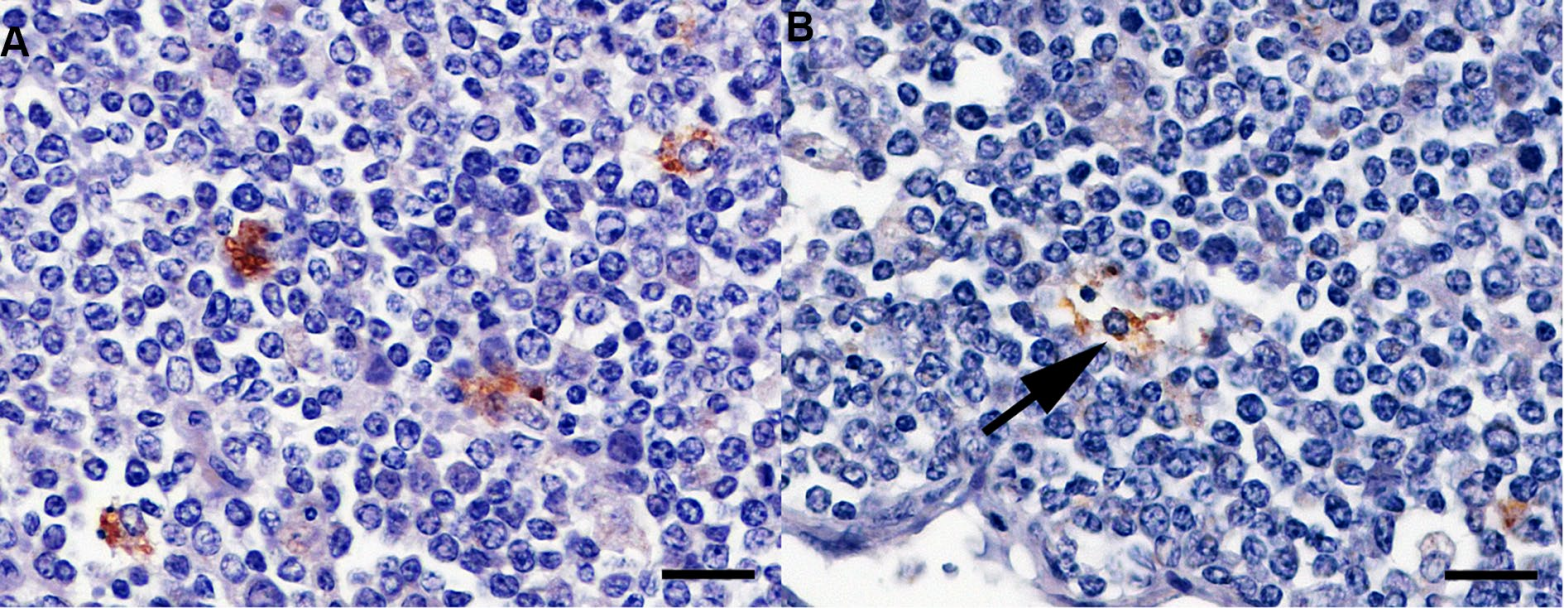
**Initial PrP** ^**BSE**^ **accumulation in a follicle of the IPP.** WAIT 12 (4 mpi); **A** initial accumulation characterised by a fine granular reaction pattern in the cytoplasm of individual tingible body macrophages (TBMs); **B** fine granular PrP^BSE^ depositions in a TBM (arrow); immunohistochemistry, PrP mAb 6C2, bar 20 µm.

**Figure 2:**
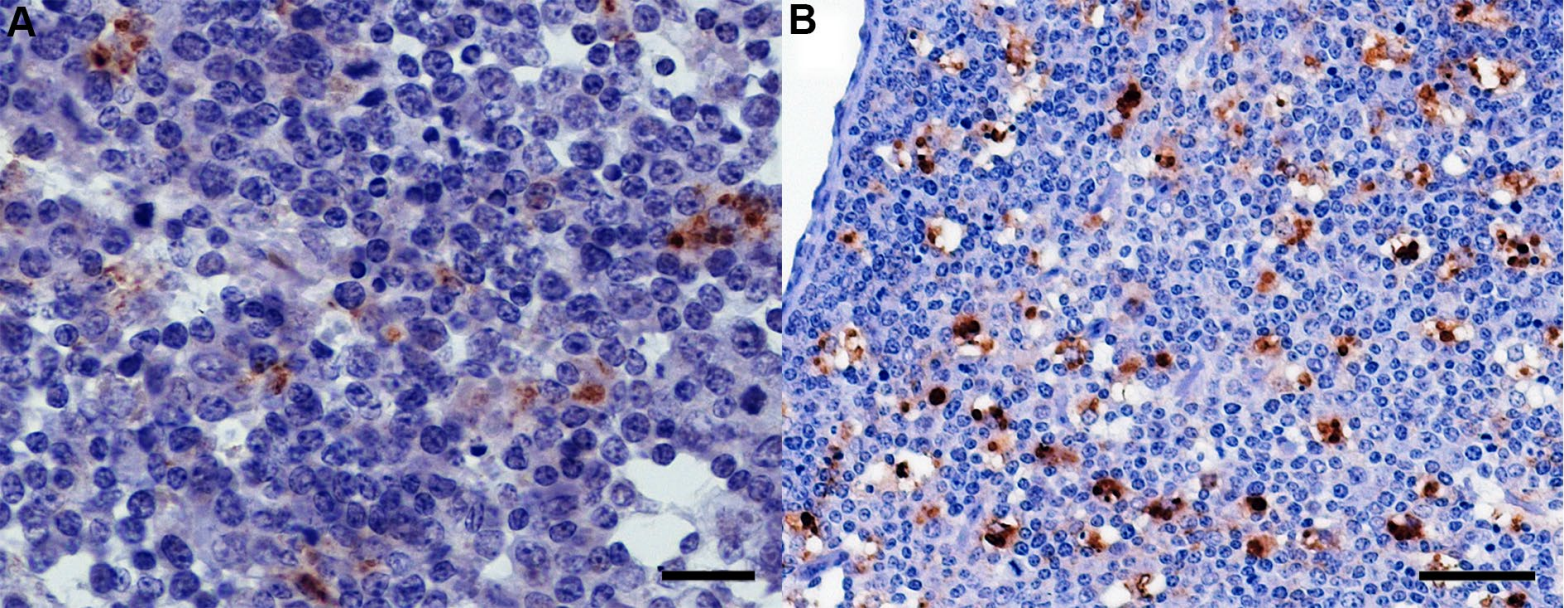
**Increased PrP** ^**BSE**^ **accumulation in IPP follicles. A** WAIT 13 (4 mpi), multiple fine and coarse granules in the cytoplasm of TBMs, bar 20 µm; **B** WAIT 08 (6 mpi), intracytoplasmatic coarse granular reaction pattern in TBMs, bar 50 µm; immunohistochemistry, PrP mAb 6C2.

**Figure 3:**
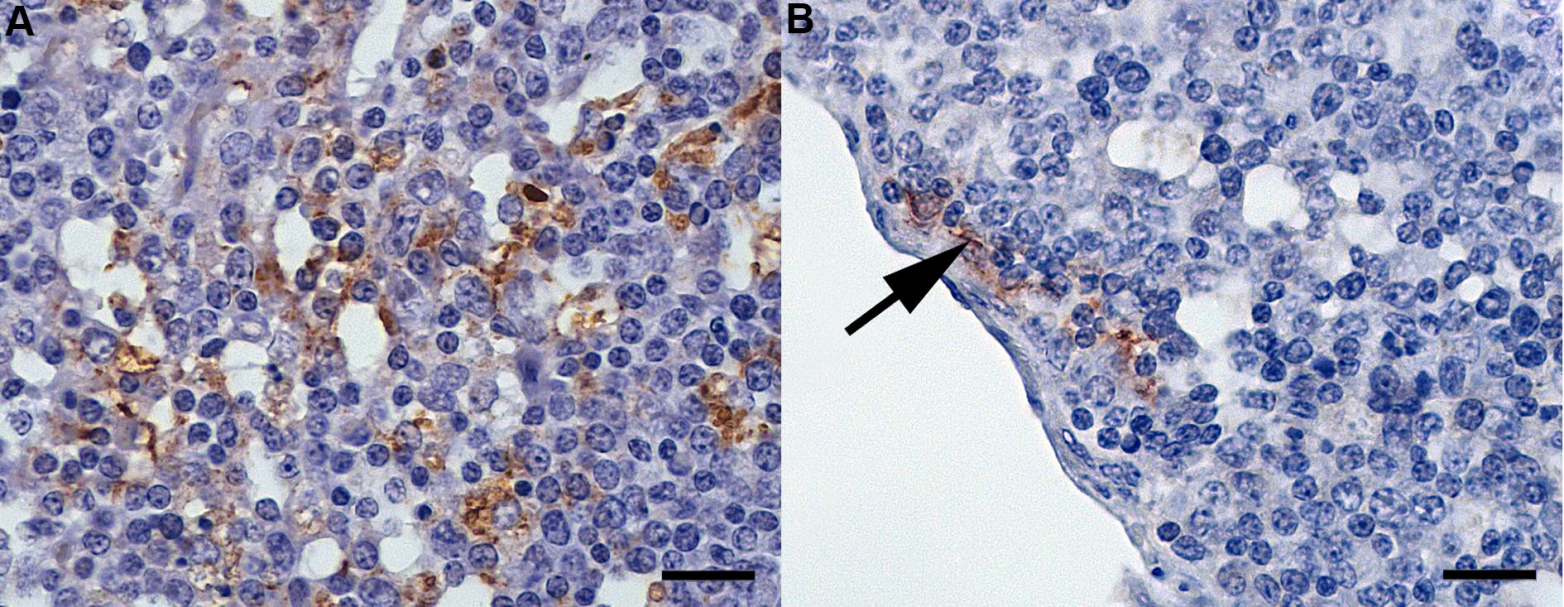
**PrP** ^**BSE**^ **accumulation in FDCs in follicles of the IPP. A** WAIT 13 (4 mpi), a net-like reaction pattern as associated with PrP^BSE^ accumulation in follicular dendritic cells (FDCs); **B** WAIT 11 (4 mpi), note the fine initiating net-like staining reaction (arrow) in the marginal zone of the follicle; immunohistochemistry, PrP mAb 6C2, bar 20 µm.

Although cellular PrP^BSE^ accumulation patterns were independent of the time points after infection, they were associated with the percentage of PrP^BSE^-positive cells per follicle (Table 2). Initial PrP^BSE^ accumulations were characterised by fine granules in one or only a few tingible body macrophages (TBMs) (Figure 1), as seen in calves with up to 0.5% of positive follicles. Increasing PrP^BSE^ accumulations were observed as multiple fine (Figure 2A) to coarse granules (Figure 2B) in several to numerous cells of one follicle, frequently observed in the animals with > 0.84% positive follicles. However, a fine as well as coarse granular or globular reaction pattern was seen in most calves with positive follicles (Table 2). A mild net-like staining pattern was associated with PrP^BSE^ accumulations in follicular dendritic cells (FDCs) (Figure 3A), primarily in calves with percentages over 0.84% positive follicles (WAIT 13, 14, 20; 4 mpi and WAIT 05, 08; 6 mpi). Furthermore, in one calf (WAIT 11; 4 mpi) only two positive follicles showed a weak initial net-like staining pattern (Figure 3B).

**Figure 4:**
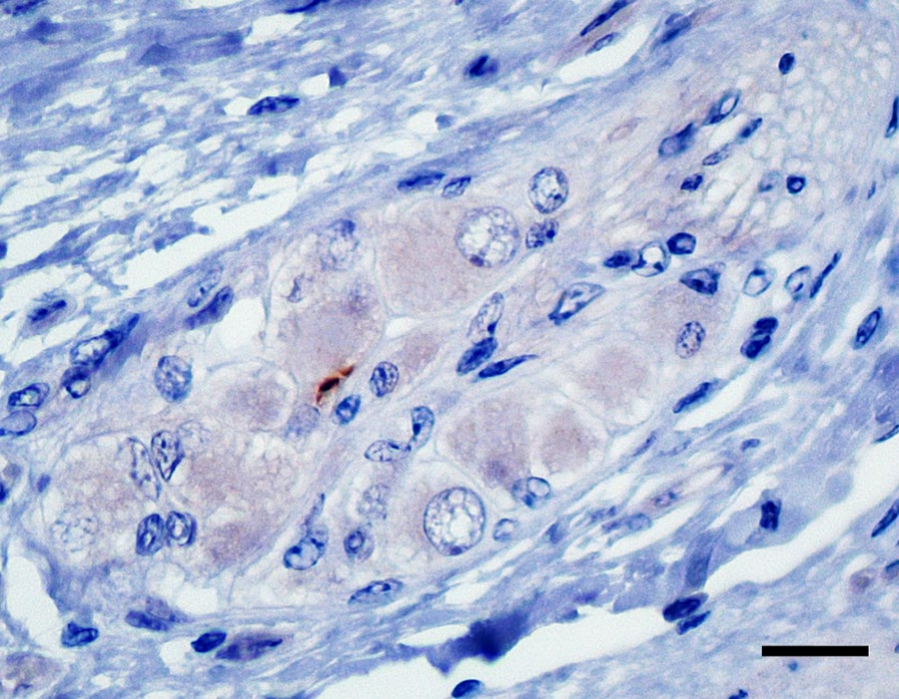
**Intraglial PrP** ^**BSE**^ **accumulation in the ENS.** WAIT 16 (2 mpi), intracellular PrP^BSE^ deposition in a glial cell in a plexus submucosus of the enteric nervous system (ENS) in the ileocaecal junction; immunohistochemistry, PrP mAb 6C2, bar 20 µm.

Interestingly, we found the enteric nervous system (ENS) to be involved in a sample of only one calf (WAIT 16; 2 mpi) in the ileocaecal junction, where a single submucosal plexus showed a positive signal, which was detectable only by mAb 6C2 (Figure 4). Here, two PrP^BSE^ granules were seen in a glial accumulation pattern, but verification was not possible by staining a reserve section with mAb F99. Besides, one positive control animal (WAIT 01; 36 mpi) had PrP^BSE^ accumulated in the myenteric plexus of the ENS.

### Tgbov XV mouse bioassay

The inoculum for the challenge of the calves had an LD_50_-titer of 10^−5.730^ (95% confidence interval, 10^−6.569^–10^−4.891^) as experimentally determined by end-point titration in Tgbov XV mice.

Bovine spongiform encephalopathy infectivity was detected in ileal Peyer’s patches (IPP) of all BSE infected calves at all examined time points (Table 1). At the time of writing, bioassays have not yet been completed for all IPP samples. The mean incubation times of between 319 and 401 days post-infection (dpi) determined for the groups of mice inoculated with IPP samples from cattle sacrificed between 4 and 8 months post infection—where the experiment has already been completed—indicates infectivity titres in the samples between 10^4.4^ ID_50_ g^−1^ (≈ 280 days) and 10^3^ ID_50_ g^−1^ (≈ 400 days), according to earlier Tgbov XV bioassay studies [20, 25]. However, for some of the samples from the 4 mpi group, low attack rates have been observed so far, and individual mice were sacrificed at 419 dpi (WAIT 13, 19) and 567 dpi (WAIT 11) on average. Interestingly, also the IPPs of the calves sacrificed at 2 months (WAIT 15 and 16) and even at 1 week (WAIT 17 and 18) post challenge contained BSE infectivity. Even though, the determined attack rates and mean incubation times of mice inoculated with those IPPs, indicate low titres of less than 10^2.5^ ID_50_ g^−1^ (in correlation to earlier Tgbov XV experiments [20, 25]).

### Protein misfolding cyclic amplification (PMCA)

**Figure 5:**
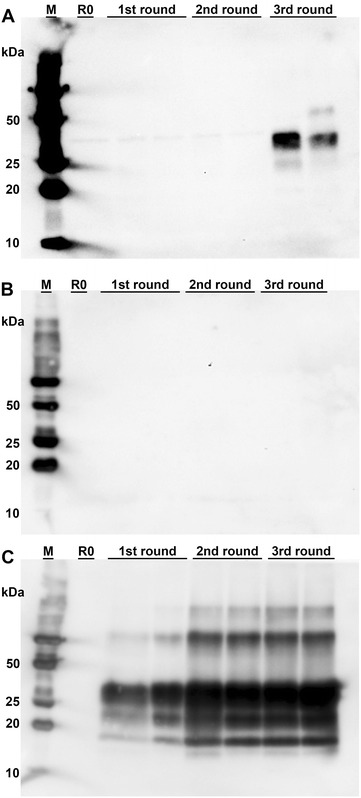
**PrP** ^**BSE**^ **amplification by PMCA in IPP samples.** PMCA detected PrP^BSE^ in the IPP of calves starting from 2 mpi; each sample was analysed in duplicate and subjected to three rounds of PMCA; M: marker, R0: analyte homogenate diluted 1:10 in Tgbov XV brain substrate without sonication; **A** WAIT 15 (2 mpi), PrP^BSE^ was detectable in the IPP of one calf as early as 2 months after oral challenge; **B** WAIT 11 (4 mpi), one IPP of a calf 4 mpi revealed a negative result; **C** WAIT 03 (8 mpi), all three PMCA rounds are positive for PrP^BSE^.

Positive results were also obtained by PMCA for the IPP of 15 out of the 18 calves that were necropsied between 1 week and 8 months post challenge (Table 1). Interestingly, there were indications for time-dependent PrP^BSE^ amplification patterns (Additional file 2). Interestingly, PrP^BSE^ was already detectable in the IPP sample of one animal that had been sacrificed as early as 2 mpi (WAIT 15), as demonstrated by a + positive reaction in the third PMCA round (Figure 5), while the second 2 mpi animal (WAIT 16) remained negative. At 4 mpi the IPP of one animal (WAIT 11) was negative (Figure 5), whereas the IPP samples of the other animals sacrificed at this time point showed positive reactions of + (WAIT 13 and 19), +(+) (WAIT 12)/++ (WAIT 14, 20) as well as ++(+) (WAIT 20). At 6 mpi, one animal was determined as + positive (WAIT 10), while the five remaining animals gave results of at least ++ positive (WAIT 05, 06, 07, 08, 09). For calves sacrificed at 8 mpi (WAIT 02, 03) as well as the positive control animals WAIT 04 (35 mpi) and WAIT 01 (36 mpi), positive results of at least ++(+) were obtained.

## Discussion

In this study, we have orally dosed young unweaned calves of 4–6 weeks of age with BSE-infected brainstem homogenate and examined the ileal Peyer’s patch (IPP), as the known major entry port of BSE prions, at earlier time points than in previous studies [2, 3, 5, 6] of 1 week as well as 2, 4, 6 and 8 months following exposure. We were able to show that PrP^BSE^ accumulation/BSE infectivity can be detected in the IPP from 1 week post infection by Tgbov XV mouse bioassay, 2 mpi by PMCA and 4 mpi by immunohistochemistry. The here presented detection of infectivity and PrP^BSE^ (by PMCA) from 2 months after oral challenge document the earliest time point of an amplification of BSE prions in bovines reported so far.

We were able to detect infectivity at 1 week post infection by transgenic mouse bioassay. Using the same transgenic mouse line Tgbov XV, in our earlier study on 4–6 months old animals, an IPP sample obtained 1 month post challenge was free of infectivity, while the samples collected 4 mpi and later were positive [4]. Thus, infectivity that was detectable at 1 week post infection in this study may most likely have been the result of residual inoculum absorbed via the intestinal mucosa. Attempts were made to trace residual brainstem inoculum in the IPP of calves at 1 week pi by using an anti-myelin oligodendrocyte glycoprotein (MOG)-IHC. However, a reliable discrimination between infected and uninfected animals could not be established and this option therefore had to be dismissed. Nonetheless, based on the bioassay results we conclude that the IPP from calves at 1 week after exposure may represent a risk of prion infection, regardless whether the BSE infectivity originates from residual inoculum or newly propagated BSE infectivity.

In previous C-BSE pathogenesis studies, the challenged animals were above 4–6 months of age [3, 6, 8, 9] and infectivity as well as PrP^BSE^ was reported for IPPs from 4 mpi onwards [3–6, 27, 28]. However, in these studies, either wild-type mice [6, 27, 28] with a lower BSE susceptibility [20] have been used for the bioassay, or samples collected at 2 mpi were not included in the analysis [3, 4]. It has been assumed that there is an initial balance between prion degradation by macrophages and accumulation in follicular dendritic cells (FDCs) until the breakdown of these clearance efforts [3, 29]. As in an earlier study, the first FDC accumulation patterns in ileal Peyer’s patch follicles were observed starting from 12 mpi, this balance was assumed to maintain for at least 8 months after infection with PrP^BSE^ only detectable in tingible body macrophages (TBMs) [3]. Interestingly, we detected PrP^BSE^ in FDCs as early as 4 mpi in several animals mostly with a higher percentage of positive follicles. This early PrP^BSE^ detection in FDCs of calves from 4 mpi, suggests an age-dependent reduction in the above described clearing efforts.

PrP^BSE^ accumulation in the enteric nervous system (ENS) was described from 16 mpi in an earlier study [3]. Our detection of glial accumulation of two PrP^BSE^ granules in a single plexus submucosus of the ENS of one animal sacrificed at 2 mpi with negative follicles might be indicative of a possible direct neuroinvasion in young calves. However, this accumulation was only seen with mAb 6C2 and could not be seen by mAb F99, which might be due to the fact that two slides do not display the exact same location as the distance between them can be up to 9 µm (5 section were cut per level). Earlier studies pointed towards a simultaneous exposure of the ENS and the lymphoreticular system via nerve fibres present in the lamina propria mucosae and the suprafollicular dome [30, 31].

Although our study was not specifically designed to address the age-dependent BSE susceptibility of cattle, the earlier detection of infectivity in the Peyer’s patches of the unweaned calves at the age of 4–6 weeks at time of challenge as compared to previous challenge data in older calves may reflect indeed an elevated susceptibility. Likewise, the onset of clinical BSE-related signs at 32 and 36 mpi in the two positive control animals may indicate a shorter average incubation time as compared to average incubation times in earlier studies utilizing older calves [2, 32]. However, these relatively short incubation times should be interpreted with caution as the animal numbers are too small in order to provide proof for this conclusion.

Young animals are assumed to be particularly susceptible to a prion infection, as pointed out by challenge experiments in sheep and mice as well as an epidemiological study of cattle farms conducted in the UK [10–13]. After oral challenge ~ 24 h- and 2–3 week-old lambs were more likely to develop BSE than older sheep after weaning [10]. Moreover, susceptibility to peripheral prion infection was decreased in aged mice, as characterized by the absence of clinical signs, since an age-dependent reduction of FDCs impaired the agent accumulation in lymphoid tissues and thus neuroinvasion [11, 12]. Similar events might occur in ruminants, as ileal Peyer’s patches of young lambs were shown to have better developed FDC networks than those of adult sheep [33]. However, we also consider that this might not be the case in bovines, as the lymphoreticular system—except for the IPP—is rarely involved in BSE pathogenesis [20, 34, 35] and is discussed not being necessarily required for neuroinvasion [30]. But in spite of that, the increased uptake of macromolecules across the intestinal mucosa in young animals [36, 37] was discussed to facilitate the BSE agent uptake and thereby result in a high BSE susceptibility [10]. Indeed, incorporation of proteins rich in beta-sheet structure occurs more easily during the suckling period, as shown by an oral experimental infection of 2-week-old and 6-month-old calves with beta-amyloid protein [38]. Hence, in our unweaned calves a facilitated uptake of the BSE prion proteins in combination with an age-dependent reduction of clearing efforts seemed to trigger such early detection of the agent in relatively large quantities.

Besides the transgenic mouse bioassay, the PMCA is considered the most sensitive method for the detection of BSE prions, as shown by the comparative endpoint titrations of BSE inoculum in Tgbov XV mice and PMCA [24, 25]. This is confirmed by our results, as we observed an overall good agreement between the results obtained in the transgenic mouse bioassay and by PMCA. Observed differences between results of IHC and protein biochemical methods may also be due to the sampling of the IPPs, as formalin-fixed IPP (for IHC) and frozen IPP (examined by PMCA and mouse bioassay) samples represent different locations of one IPP with possibly variable amounts of the BSE agent.

In summary, our study demonstrates for the first time PrP^BSE^ (by PMCA) and prion infectivity (by mouse bioassay) in the ileal Peyer’s patch (IPP) of young calves as early as 2 months after infection. From 4 mpi nearly all calves showed PrP^BSE^ positive IPP follicles (by IHC), even with PrP^BSE^ accumulation detectable in FDCs in some animals. Finally, our results confirm the IPP as the early port of entry for the BSE agent and a site of initial propagation of PrP^BSE^ and infectivity during the early pathogenesis of the disease. Therefore, our study supports the recommendation to remove the last four metres of the small intestine (distal ileum) at slaughter, as designated by current legal requirements for countries with a controlled BSE risk status, as an essential measure for consumer and public health protection.

## Additional files

**Additional file 1.** **PrP** ^**BSE**^ **accumulation in the obex and ileal Peyer’s patch of positive control cattle.** WAIT 01 showed moderate and WAIT 04 intense PrP^BSE^ accumulation in the brainstem. PrP^BSE^ was present in the follicles of both animals ileal Peyer’s patches (IPP) as well as in enteric nervous system (ENS) of the IPP of WAIT 01. **A:** moderate fine to coarse granular PrP^BSE^ accumulation in the neuropil and in the cytoplasm of neurons of the dorsal motor nucleus of the vagus (DMNV) in the obex of WAIT 01 (36 mpi), bar 50 µm; **B:** intense extra- and intracellular coarse granular staining reaction in the neuropil and neurons of the DMNV of WAIT 04 (35 mpi), bar 50 µm; **C:** net-like staining reaction in an IPP follicle of WAIT 01, bar 50 µm; **D:** neuron of the myenteric plexus of the ENS with intracytoplasmatic staining reaction, bar 20 µm, WAIT 01 (36 mpi); **E:** coarse granular PrP^BSE^ accumulation in an IPP follicle of WAIT 04, bar 50 µm; A – E: Immunohistochemistry, PrP mab 6C2; **F:** PrP^BSE^ amplification by PMCA in the IPP of WAIT 01; **G:** PMCA results for IPP of WAIT 04; F- G: M: marker, R0: analyte homogenate diluted 1:10 in Tgbov XV brain substrate without sonication.

**Additional file 2.** **Results of PMCA analyses of the ileal Peyer’s patch samples for the rest of calves.** The quantity of PrP^BSE^ detectable by PMCA was observed to correlate with the time point after infection. All analyte tissue samples were analysed in duplicate and subjected to three rounds of PMCA. M: marker, R0: analyte homogenate diluted 1:10 in Tgbov XV brain substrate without sonication.

## References

[1] Prusiner SB (1998). Prions. Proc Natl Acad Sci U S A.

[2] Kaatz M, Fast C, Ziegler U, Balkema-Buschmann A, Hammerschmidt B, Keller M, Oelschlegel A, McIntyre L, Groschup MH (2012). Spread of classic BSE prions from the gut via the peripheral nervous system to the brain. Am J Pathol.

[3] Hoffmann C, Eiden M, Kaatz M, Keller M, Ziegler U, Rogers R, Hills B, Balkema-Buschmann A, van Keulen L, Jacobs JG, Groschup MH (2011). BSE infectivity in jejunum, ileum and ileocaecal junction of incubating cattle. Vet Res.

[4] Fast C, Keller M, Balkema-Buschmann A, Hills B, Groschup MH (2013). Complementary studies detecting classical bovine spongiform encephalopathy infectivity in jejunum, ileum and ileocaecal junction in incubating cattle. Vet Res.

[5] Stack MJ, Moore SJ, Vidal-Diez A, Arnold ME, Jones EM, Spencer YI, Webb P, Spiropoulos J, Powell L, Bellerby P, Thurston L, Cooper J, Chaplin MJ, Davis LA, Everitt S, Focosi-Snyman R, Hawkins SAC, Simmons MM, Wells GAH (2011). Experimental bovine spongiform encephalopathy: detection of PrP^Sc^ in the small intestine relative to exposure dose and age. J Comp Pathol.

[6] Terry LA, Marsh S, Ryder SJ, Hawkins SAC, Wells GAH, Spencer YI (2003). Detection of disease-specific PrP in the distal ileum of cattle exposed orally to the agent of bovine spongiform encephalopathy. Vet Rec.

[7] Carlens O (1928). Studien über das lymphatische Gewebe des Darmkanals bei einigen Haustieren, mit besonderer Berücksichtigung der embryonalen Entwicklung, der Mengenverhältnisse und der Altersinvolution dieses Gewebes im Dünndarm des Rindes. Zeitschrift für Anatomie und Entwicklungsgeschichte.

[8] Hoffmann C, Ziegler U, Buschmann A, Weber A, Kupfer L, Oelschlegel A, Hammerschmidt B, Groschup MH (2007). Prions spread via the autonomic nervous system from the gut to the central nervous system in cattle incubating bovine spongiform encephalopathy. J Gen Virol.

[9] Wells GAH, Dawson M, Hawkins SAC, Austin AR, Green RB, Dexter I, Horigan MW, Simmons MM, Gibbs CJ (1996). Preliminary observations on the pathogenesis of experimental bovine spongiform encephalopathy. Bovine spongiform encephalopathy: the BSE dilemma.

[10] Hunter N, Houston F, Foster J, Goldmann W, Drummond D, Parnham D, Kennedy I, Green A, Stewart P, Chong A (2012). Susceptibility of young sheep to oral infection with bovine spongiform encephalopathy decreases significantly after weaning. J Virol.

[11] Brown KL, Mabbott NA (2014). Evidence of subclinical prion disease in aged mice following exposure to bovine spongiform encephalopathy. J Gen Virol.

[12] Brown KL, Wathne GJ, Sales J, Bruce ME, Mabbott NA (2009). The effects of host age on follicular dendritic cell status dramatically impair scrapie agent neuroinvasion in aged mice. J Immunol.

[13] Arnold ME, Wilesmith JW (2004). Estimation of the age-dependent risk of infection to BSE of dairy cattle in Great Britain. Prev Vet Med.

[14] Braun U, Kihm U, Pusterla N, Schonmann M (1997). Clinical examination upon suspicion of bovine spongiform encephalopathy (BSE). Schweiz Arch Tierheilkd.

[15] Hardt M, Baron T, Groschup MH (2000). A comparative study of immunohistochemical methods for detecting abnormal prion protein with monoclonal and polyclonal antibodies. J Comp Pathol.

[16] O’Rourke KI, Baszler TV, Besser TE, Miller JM, Cutlip RC, Wells GAH, Ryder SJ, Parish SM, Hamir AN, Cockett NE, Jenny A, Knowles DP (2000). Preclinical diagnosis of scrapie by immunohistochemistry of third eyelid lymphoid tissue. J Clin Microbiol.

[17] Spraker TR, O’Rourke KI, Balachandran A, Zink RR, Cummings BA, Miller MW, Powers BE (2002). Validation of monoclonal antibody F99/97.6.1 for immunohistochemical staining of brain and tonsil in mule deer (*Odocoileus hemionus*) with chronic wasting disease. J Vet Diagn Investig.

[18] Krasemann S, Groschup MH, Harmeyer S, Hunsmann G, Bodemer W (1996). Generation of monoclonal antibodies against human prion proteins in PrP0/0 mice. Mol Med.

[19] Kascsak RJ, Rubenstein R, Merz PA, Tonna-DeMasi M, Fersko R, Carp RI, Wisniewski HM, Diringer H (1987). Mouse polyclonal and monoclonal antibody to scrapie-associated fibril proteins. J Virol.

[20] Buschmann A, Groschup MH (2005). Highly bovine spongiform encephalopathy-sensitive transgenic mice confirm the essential restriction of infectivity to the nervous system in clinically diseased cattle. J Infect Dis.

[21] Gretzschel A, Buschmann A, Eiden M, Ziegler U, Luhken G, Erhardt G, Groschup MH (2005). Strain typing of German transmissible spongiform encephalopathies field cases in small ruminants by biochemical methods. J Vet Med B Infect Dis Vet Public Health.

[22] R Core Team (2012) R: a language and environment for statistical computing. R Foundation for Statistical Computing, Vienna. ISBN 3-900051-07-0. http://www.R-project.org/

[23] Højsgaard S, Halekoh U with contributions from Robison-Cox J, Wright K, Leidi AA (2012) doBy: doBy-Groupwise summary statistics, general linear contrasts, population means (least-squares-means), and other utilities. R package version 4.5-5. http://CRAN.R-project.org/package=doBy

[24] Franz M, Eiden M, Balkema-Buschmann A, Greenlee J, Schatzl H, Fast C, Richt J, Hildebrandt JP, Groschup MH (2012). Detection of PrP^Sc^ in peripheral tissues of clinically affected cattle after oral challenge with bovine spongiform encephalopathy. J Gen Virol.

[25] Balkema-Buschmann A, Eiden M, Hoffmann C, Kaatz M, Ziegler U, Keller M, Groschup MH (2011). BSE infectivity in the absence of detectable PrP^Sc^ accumulation in the tongue and nasal mucosa of terminally diseased cattle. J Gen Virol.

[26] Buschmann A, Pfaff E, Reifenberg K, Müller HM, Groschup MH (2000). Detection of cattle-derived BSE prions using transgenic mice overexpressing bovine PrP^C^. Arch Virol Suppl.

[27] Wells GAH, Dawson M, Hawkins SAC, Green RB, Dexter I, Francis ME, Simmons MM, Austin AR, Horigan MW (1994). Infectivity in the ileum of cattle challenged orally with bovine spongiform encephalopathy. Vet Rec.

[28] Arnold ME, Hawkins SAC, Green R, Dexter I, Wells GAH (2009). Pathogenesis of experimental bovine spongiform encephalopathy (BSE): estimation of tissue infectivity according to incubation period. Vet Res.

[29] Mabbott NA, Bruce ME (2001). The immunobiology of TSE diseases. J Gen Virol.

[30] Jeffrey M, González L, Espenes A, Press CM, Martin S, Chaplin M, Davis L, Landsverk T, MacAldowie C, Eaton S, McGovern G (2006). Transportation of prion protein across the intestinal mucosa of scrapie-susceptible and scrapie-resistant sheep. J Pathol.

[31] Heggebø R, González L, Press CM, Gunnes G, Espenes A, Jeffrey M (2003). Disease-associated PrP in the enteric nervous system of scrapie-affected Suffolk sheep. J Gen Virol.

[32] Arnold ME, Ryan JBM, Konold T, Simmons MM, Spencer YI, Wear A, Chaplin M, Stack M, Czub S, Mueller R, Webb PR, Davis A, Spiropoulos J, Holdaway J, Hawkins SAC, Austin AR, Wells GAH (2007). Estimating the temporal relationship between PrP^Sc^ detection and incubation period in experimental bovine spongiform encephalopathy of cattle. J Gen Virol.

[33] Marruchella G, Ligios C, Di Guardo G (2012). Age, scrapie status, PrP genotype and follicular dendritic cells in ovine ileal Peyer’s patches. Res Vet Sci.

[34] Wells GAH, Spiropoulos J, Hawkins SAC, Ryder SJ (2005). Pathogenesis of experimental bovine spongiform encephalopathy: preclinical infectivity in tonsil and observations on the distribution of lingual tonsil in slaughtered cattle. Vet Rec.

[35] Espinosa JC, Morales M, Castilla J, Rogers M, Torres JM (2007). Progression of prion infectivity in asymptomatic cattle after oral bovine spongiform encephalopathy challenge. J Gen Virol.

[36] Udall JN, Walker WA (1982). The physiologic and pathologic basis for the transport of macromolecules across the intestinal tract. J Pediatr Gastroenterol Nutr.

[37] Udall JN, Pang K, Fritze L, Kleinman R, Walker WA (1981). Development of gastrointestinal mucosal barrier. I. The effect of age on intestinal permeability to macromolecules. Pediatr Res.

[38] Ano Y, Nakayama H, Sakai Y, Sakudo A, Endo M, Ebisu S, Li J, Uetsuka K, Manabe N, Onodera T (2008). Incorporation of beta-amyloid protein through the bovine ileal epithelium before and after weaning: model for orally transmitted amyloidoses. Microbiol Immunol.

