# Supplementary figures and images for: Detection of PrPBSE and prion infectivity in the ileal Peyer’s patch of young calves as early as 2 months after oral challenge with classical bovine spongiform encephalopathy
Source: Vet Res. 2017 Dec 19;48:88. doi: 10.1186/s13567-017-0495-5 (PMC5738053; doi:10.1186/s13567-017-0495-5)

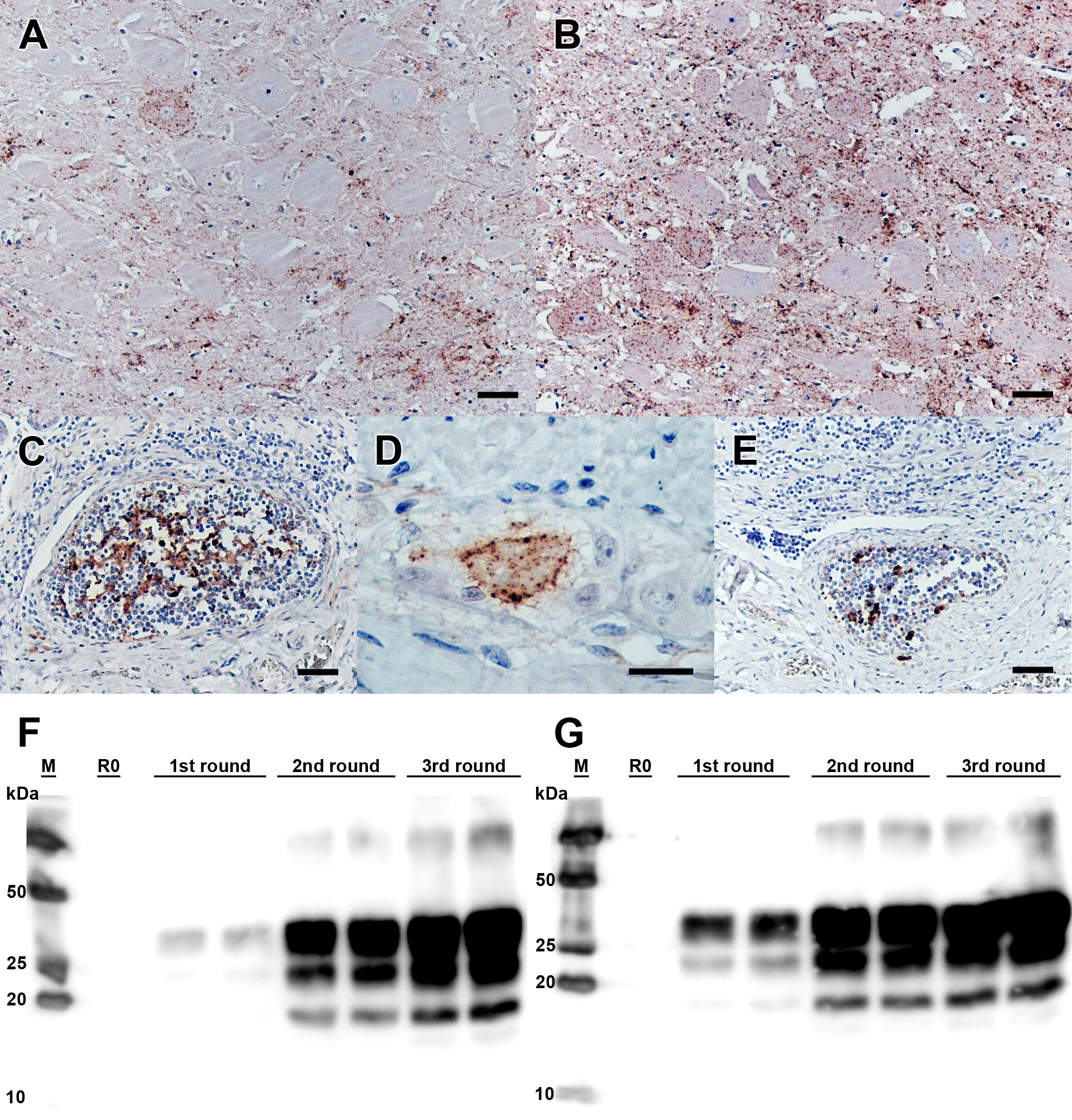

Supplement: Supplementary file 1 — Additional file 1. PrP BSE accumulation in the obex and ileal Peyer’s patch of positive control cattle. WAIT 01 showed moderate and WAIT 04 intense PrPBSE accumulation in the brainstem. PrPBSE was present in the follicles of both animals ileal Peyer’s patches (IPP) as well as in enteric nervous system (ENS) of the IPP of WAIT 01. A: moderate fine to coarse granular PrPBSE accumulation in the neuropil and in the cytoplasm of neurons of the dorsal motor nucleus of the vagus (DMNV) in the obex of WAIT 01 (36 mpi), bar 50 µm; B: intense extra- and intracellular coarse granular staining reaction in the neuropil and neurons of the DMNV of WAIT 04 (35 mpi), bar 50 µm; C: net-like staining reaction in an IPP follicle of WAIT 01, bar 50 µm; D: neuron of the myenteric plexus of the ENS with intracytoplasmatic staining reaction, bar 20 µm, WAIT 01 (36 mpi); E: coarse granular PrPBSE accumulation in an IPP follicle of WAIT 04, bar 50 µm; A – E: Immunohistochemistry, PrP mab 6C2; F: PrPBSE amplification by PMCA in the IPP of WAIT 01; G: PMCA results for IPP of WAIT 04; F- G: M: marker, R0: analyte homogenate diluted 1:10 in Tgbov XV brain substrate without sonication. [file 13567_2017_495_MOESM1_ESM.tif]

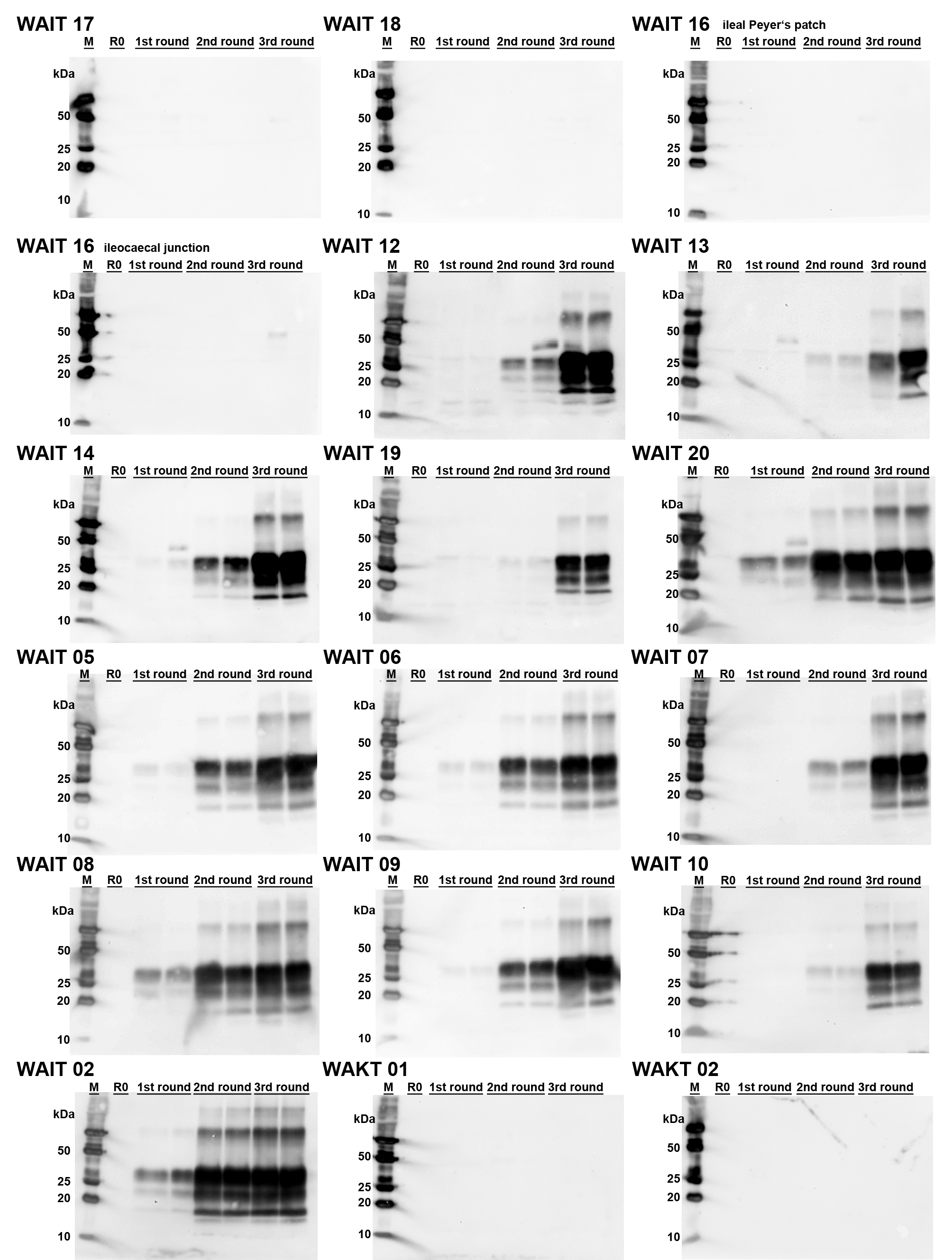

Supplement: Supplementary file 2 — Additional file 2. Results of PMCA analyses of the ileal Peyer’s patch samples for the rest of calves. The quantity of PrPBSE detectable by PMCA was observed to correlate with the time point after infection. All analyte tissue samples were analysed in duplicate and subjected to three rounds of PMCA. M: marker, R0: analyte homogenate diluted 1:10 in Tgbov XV brain substrate without sonication. [file 13567_2017_495_MOESM2_ESM.tif]
